# Stress and Refractive Index Control of SiO_2_ Thin Films for Suspended Waveguides

**DOI:** 10.3390/nano10112105

**Published:** 2020-10-23

**Authors:** Neal Wostbrock, Tito Busani

**Affiliations:** 1 Department of Nanoscience and Microsystems Engineering, School of Engineering, University of New Mexico, MSC01 1120, Albuquerque, NM 87131-0001, USA; nwostbrock@unm.edu; 2 Center for High Technology Materials (CHTM), University of New Mexico, MSC04 2710, 1313 Goddard SE, Albuquerque, NM 87106-4343, USA; 3 Department of Electrical and Computer Engineering (ECE), School of Engineering, University of New Mexico, MSC01 1100, Albuquerque, NM 87131-0001, USA

**Keywords:** air bridge, optical filter, UV, ICPVD, deposition

## Abstract

Film stress and refractive index play an important role in the fabrication of suspended waveguides. SiO_2_ waveguides were successfully fabricated on multiple substrates including Si, Ge, and Al_2_O_3_ wafers; the waveguides were deposited using inductively coupled plasma chemical vapor deposition at 100 °C. The precursor gases were SiH_4_ and N_2_O at 1:3 and 1:9 ratios with variable flow rates. The occurrence of intrinsic stress was validated through the fabrication of suspended SiO_2_ bridges, where the curvature of the bridge corresponded to measured intrinsic stress, which measured less than 1 µm thick and up to 50 µm in length. The flow rates allow film stress tunability between 50 and −65 MPa, where a negative number indicates a compressive state of the SiO_2_. We also found that the gas ratios have a slight influence on the refractive index in the UV and visible range but do not affect the stress in the SiO_2_ bridges. To test if this method can be used to produce multi-layer devices, three layers of SiO_2_ bridges with air cladding between each bridge were fabricated on a silicon substrate. We concluded that a combination of low temperature deposition (100 °C) and photoresist as the sacrificial layer allows for versatile SiO_2_ bridge fabrication that is substrate and refractive index independent, providing a framework for future tunable waveguide fabrication.

## 1. Introduction

Suspended SiO_2_ structures are important for a number of optical and photonic integrated circuit (PIC) applications such as broad spectra frequency combs [1], low propagation loss waveguides [2], and UV–visible optical filters [3]. In addition to these applications, suspended waveguides can be applied to UV absorption spectroscopy and an emerging class of gallium nitride (GaN) nanowire-based photonic devices that could benefit from UV transparent waveguides—including near-field scanning optical microscopy (NSOM), vertical cavity lasers, and direct write lithography [4,5]. In a GaN nanowire-based application, a waveguide could be used to optically pump the nanowire for emission and collect the light for signal analysis. There have been recent advances in the fabrication of GaN nanowires for these optical and mechanical applications, however, a low-loss photonic integrated circuit to propagate light to and from GaN nanowires has not yet been developed [6]. The waveguides need to operate in the UV spectrum (less than 365 nm) due to the high band gap of GaN (3.4 eV) and be fabricated on a GaN substrate to conform to nanowire fabrication. Common low-loss waveguide materials in the UV range include Si_3_N_4_, Al_2_O_3_, AlN, and SiO_2_; however, Si_3_N_4_, Al_2_O_3_, and AlN all have non-zero absorption coefficients at 266 nm, a common pumping wavelength [7,8,9]. Using SiO_2_ as the waveguide material and air as the cladding in these devices allows for the combination of a low absorption coefficient material and a high refractive index contrast enabling further enhancement and efficiency [10]. A SiO_2_ bridge is necessary to create air cladding on both the top and the bottom of the waveguide, however, suspended structures may be susceptible to stress relaxation during fabrication when the scaffold material is removed and may buckle or lift, depending on either compressive or tensile intrinsic stresses, respectively [11]. Therefore, controlling the residual stress of the suspended material is important to achieve the desired structure.

There have been multiple approaches to the deposition or growth of a SiO_2_ film for optical and PIC devices while controlling for the stress of the deposited films. One approach is to use a thermal oxide to achieve a low-stress suspended SiO_2_ structure [12]. Considering that a thermal oxide is grown at a high temperature and requires a silicon layer for growth, this approach is not compatible with devices requiring low temperature fabrication or no silicon layers. Plasma-enhanced chemical vapor deposition (PECVD) has also been used with post-deposition annealing at 600 °C to achieve a film with tensile stress [13]. Intrinsic stress can also be controlled by varying the reactive gas ratios [14]. However, controlling the intrinsic stress by changing the deposition or growth parameters can inadvertently affect the refractive index, an important consideration for the application as a waveguiding material. Ideally, a SiO_2_ bridge requires a low intrinsic stress to fabricate efficient and reliable waveguides. Furthermore, tunable intrinsic stress might serve broader applications.

The focus of this paper was to investigate the role of N_2_O and SiH_4_ gas flowrate on the residual stress and refractive index of SiO_2_ films deposited by inductively coupled plasma chemical vapor deposition (ICPCVD) for different N_2_O to SiH_4_ gas ratios. The effects of individual gas flowrates on stress and refractive index have been studied, however, a method for tuning the stress while keeping the refractive index constant was not determined [15,16]. While N_2_O and SiH_4_ gas ratios have also been studied, deposition gas flowrates have not been studied independently from gas ratios to the best of our knowledge [17,18,19]. To gain a clear understanding, we also demonstrated the control of these material properties by fabricating SiO_2_ bridges and discussing how it might improve the process development for devices requiring suspended SiO_2_ structures.

## 2. Materials and Methods

SiO_2_ was deposited on 2″ Si <100> wafers with a Trion Orion III ICPCVD at conditions with constant temperature, pressure, deposition time, and RF power: 100 °C, 10 mtorr, 500 s, and 100 W, respectively. Stress measurements were taken from films created with either one of two deposition gas ratios, 1:9 or 1:3 SiH_4_:N_2_O, and variable flowrates. Flowrates for the 1:9 gas ratio ranged from 2:18 to 6:54 sccm while the flowrates for the 1:3 gas ratio ranged from 2:6 to 6:18 sccm. Before the deposition of SiO_2_, initial measurements were taken to establish the baseline thickness and curvature of 2″ Si <100> wafers to be used as substrates for the SiO_2_ films. A Flexus F2320 stress measurement system and dial indicator were used to measure the curvature and thickness of the substrate. After deposition, film thickness and refractive index measurements were initially measured with a Filmetrics F50 reflectometer and the measurements were verified with a spectroscopic J.A. Woollam Ellipsometer for the precise fitting of thickness and refractive index. The substrates were then re-scanned with the Flexus F2320 stress measurement system to calculate the stress of the film based on initial substrate curvature, final substate curvature, substrate thickness, substrate mechanical properties, and film thickness, i.e., Stoney’s equation [20].

After the stress measurements of the films were taken, suspended SiO_2_ bridges were fabricated on silicon (Si), germanium (Ge), and sapphire (Al_2_O_3_) substrates using the same parameters as described above, except controlling the film thickness instead of deposition time. Bare wafers were diced into 10 mm square samples. The wafers were cleaned with acetone and iso-propyl alcohol then dehydrated at 180 °C for 5 min. The photoresist solution, five parts AZ5214 to four parts EBR 10-A, was spun with the samples at 5000 rpm for a target thickness of 350 nm and post-baked for 60 s at 90 °C. The photoresist was patterned with a contact mask composed of 2 mm-long lines with widths varying in the range of 10–50 µm in 5 µm increments. The samples were developed, rinsed, and blown dry with N_2_, then post-baked on a hot plate at 110 °C for 90 s. A 750 nm-thick SiO_2_ film was deposited over the patterned samples using the ICPCVD deposition conditions described above. For stacked layers, the previous steps were repeated for each additional bridge and a Karl Suss MA6 mask aligner was used for the alignment of the bridges. The samples were cleaved transverse to the patterned lines. The sacrificial photoresist scaffold was removed with a 30 min plasma exposure of O_2_, CO_2_, and N_2_O gases with a platen temperature of 110 °C. The samples were then sputter coated in Au and imaged on a Nova NanoSEM 450 instrument.

## 3. Results

**Figure 1:**
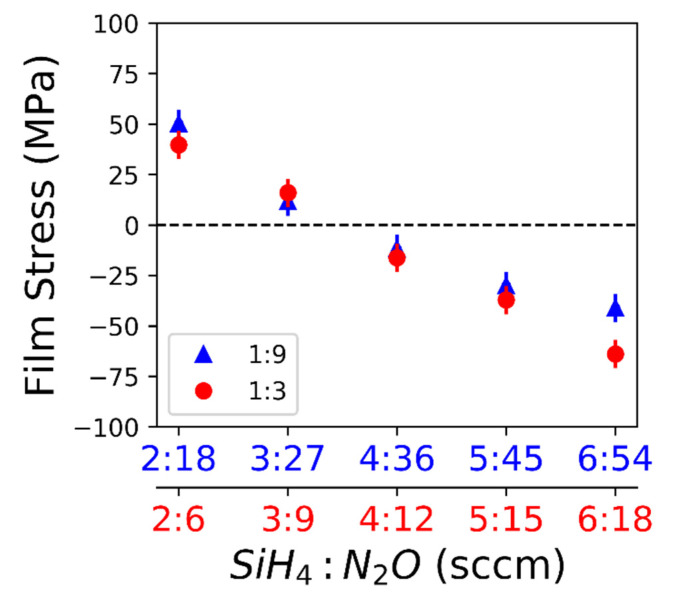
Residual SiO_2_ film stress varies with flowrate of SiH_4_ and N_2_O. A positive value in film stress indicates a tensile film and a negative value indicates a compressive film. For the 1:9 SiH_4_ to N_2_O ratio (triangle) and 1:3 SiH_4_ to N_2_O ratio (circle), the maximum standard deviation of the measurement is 6.4 MPa.

Figure 1 shows that the films deposited with lower flowrates consistently produced a film with a tensile residual stress whereas higher flowrates produced films with a compressive residual stress.

**Figure 2:**
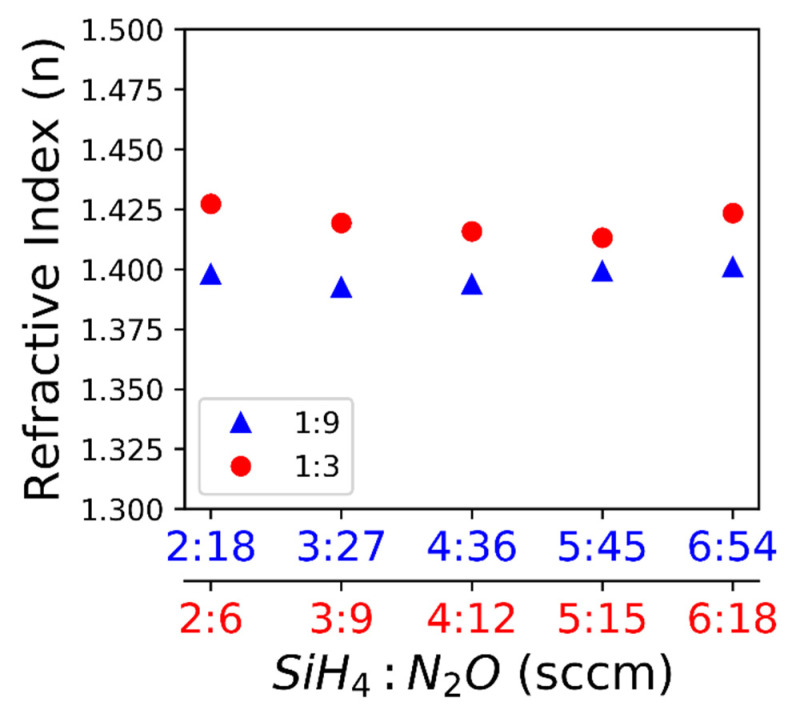
The measured refractive index is shown with respect to the flowrate: The refractive index is measured by ellipsometry (error < 0.005).

The highest tensile stress measured was 50.1 MPa, made using a flowrate of 2:18 sccm SiH_4_:N_2_O. Transition from the tensile to compressive stress for both gas ratios occurred at 3–4 sccm of SiH_4_. The maximum compressive stress measured was −63.9 MPa made using a flowrate of 6:18 sccm SiH_4_:N_2_O. Stress in the films made using either the 1:9 or 1:3 gas ratios did not vary significantly except for the highest flowrate, 6 sccm SiH_4_. Deviation from a linear regression at 6 sccm SiH_4_ could mean that the plasma was unstable with a high N_2_O gas concentration. The flowrate appears to affect the stress created during film deposition but the porosity or stoichiometry changes, resulting from the different flowrates, may affect the refractive index. Therefore, the refractive index of each sample was measured to ensure that only the film stress was changing with flowrate, not the porosity or stoichiometry. Figure 2 shows that the refractive index only changes with different flow ratios but the refractive index does not change with flowrate for a given flow ratio. Therefore, the variation of stress within a flow ratio cannot be attributed to a change in porosity or stoichiometry.

**Figure 3:**
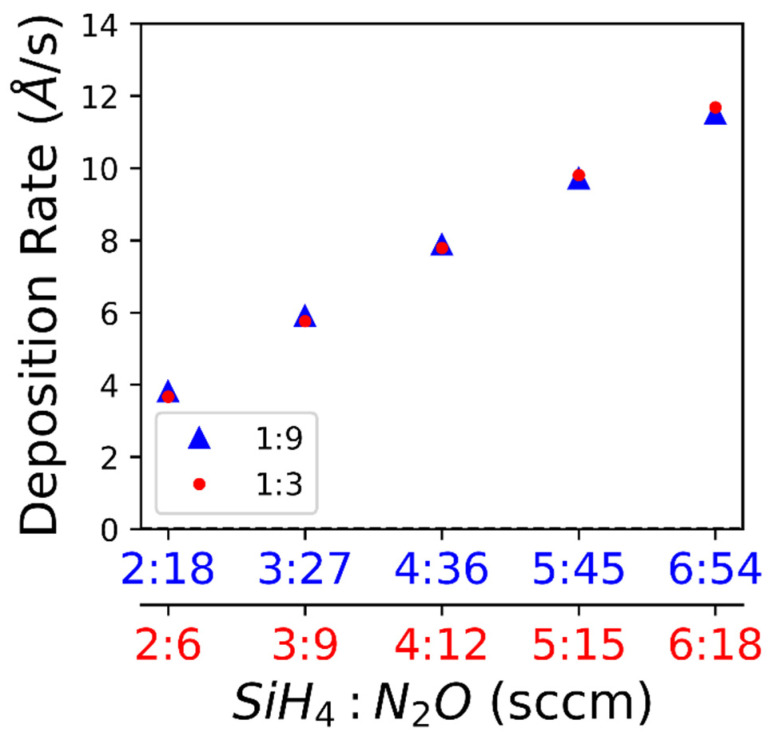
The deposition rate shown with respect to the flowrate. The deposition rate of the films was calculated using the thickness measurements from the ellipsometry data and verified with SEM cross-section measurements.

Fine-tuning the intrinsic stress during film deposition without affecting the refractive index appears feasible. The low refractive index (n) values compared to bulk SiO2 (n = 1.5) indicate that the porosity or stoichiometry may be different from bulk SiO2. The deposition rate varies with flowrate, as shown in Figure 3.

## 4. Discussion

We observed a transition from the tensile stress to compressive stress with increasing deposition gas flowrates. The deposition rate increases with increasing flow rate, which can be explained by more reagents reaching the substrate surface and the deposition not being reaction limited [21]. Since the deposition temperature is held constant throughout all samples, residual stress due to a coefficient of thermal expansion (CTE) mismatch can be refuted. Additionally, the films were deposited on various substrates showing that the mechanism was not likely substrate specific. In some materials, such as Cu, the film thickness directly effects film stress, however it has been shown that CVD SiO_2_ has no such effect [22,23]. There has been a distinction drawn between CVD, typically yielding tensile films due to random deposition and gradual cross linking, and plasma CVD, typically yielding compressive films due to energetic bombardment and rapid growth [24,25]. A kinetic modeling study, using a dimensionless D/RL parameter where D is effective diffusivity, L is grain size, and R is the deposition rate, predicts a compressive film for a large D/RL value and a tensile film for a low D/RL value; however, this is not consistent with our results in terms of deposition rate [26]. In our study, the effective diffusivity is likely constant since the deposition temperature and pressure are held constant. Additionally, in this study, the refractive index is constant for a given deposition gas ratio (Figure 2), indicating that the porosity of the films does not vary significantly [27]. For comparison, the deposition gas ratios have a significant effect on the optical properties of SiN films probably due to a change in stoichiometry [28]. One study looking at SiC films reports an increasing compressive stress with increased power and deposition rate which they attribute to densification of the film but report no significant change in the refractive index [29]. There are many variables that can affect stress in a deposited film. In our study, grain size could be the major contributor to the stress transition from tensile to compressive with increasing deposition gas flow rates. Future research will examine the difference between the kinetic model [26] and the results of this study.

**Figure 4:**
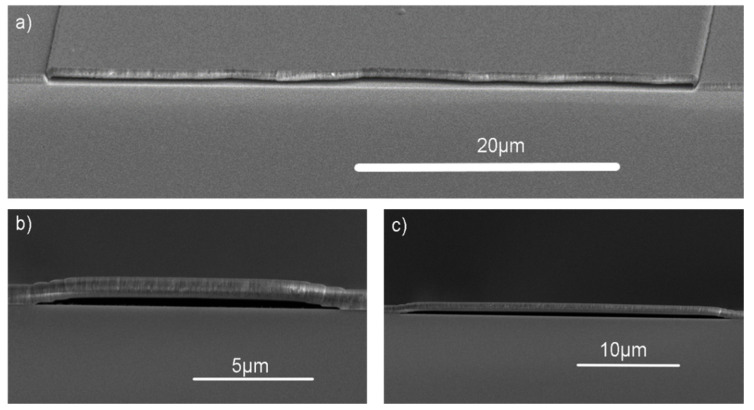
SEM images were taken of the suspended structures of various bridge lengths. Bridge lengths up to 50 µm were fabricated on Si with a SiO_2_ thickness of 750 nm: (**a**) 50 µm bridge; (**b**) 10 µm bridge; and (**c**) 30 µm bridge.

We examined how intrinsic stress affects the fabrication of suspended SiO_2_ bridges (hereafter referred to as ‘bridges’) and verified stress measurements by observing the stress relaxation and deformation of the fabricated bridges. Since there were no significant differences in intrinsic stress between the 1:3 and 1:9 gas ratios, only the 1:3 gas ratio was used for bridge fabrication. Examples of 10, 30, and 50 µm-wide bridges are shown in Figure 4 (bridges were, however, created in 5 µm increments ranging from 10 to 50 µm in width). The stress relaxation due to residual stress can be qualitatively observed by comparing the sacrificial photoresist height to the height of the bridges after the photoresist was removed.

**Figure 5:**
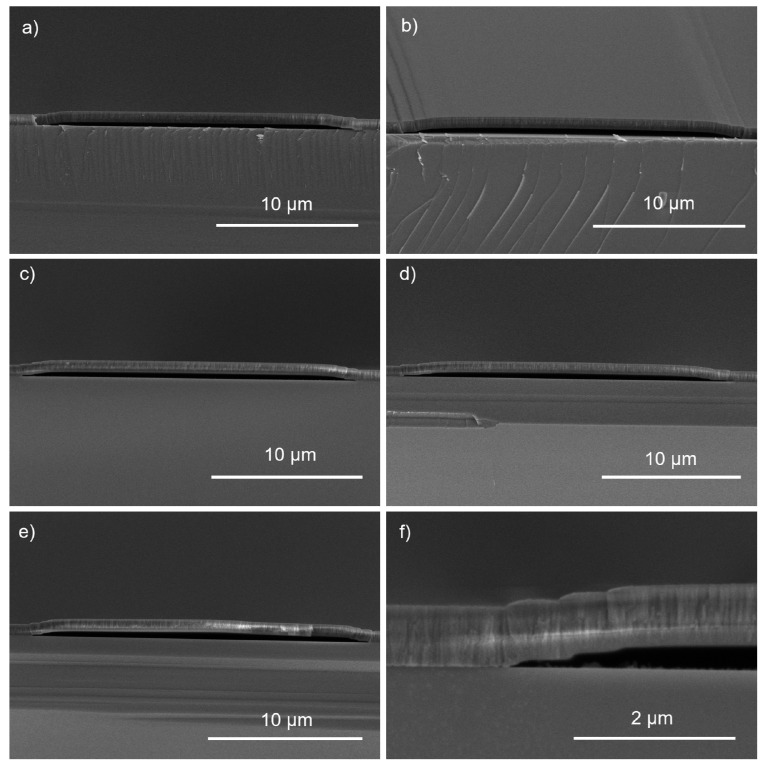
Using SEM images, the gap heights were measured from the center of the bridge to the substrate. SiH_4_ to N_2_O ratios and gap heights are reported, respectively: (**a**) 2:6 sccm and 317 nm; (**b**) 3:9 sccm and 348 nm; (**c**) 4:12 sccm and 368 nm; (**d**) 5:15 sccm and 425 nm; (**e**) 6:18 sccm and 496 nm; (**f**) close-up image of a bridge corner shows there is no cracking.

The sacrificial photoresist height was 362 nm. After the photoresist was removed, an observed increased gap height is indicative of stress relaxation in a compressive film, whereas a decreased gap height is indicative of stress relaxation in a tensile film. The gap height after the sacrificial photoresist was removed from the 20 µm-wide bridges and was measured with cross-section SEM images and shown in Figure 5.

**Figure 6:**
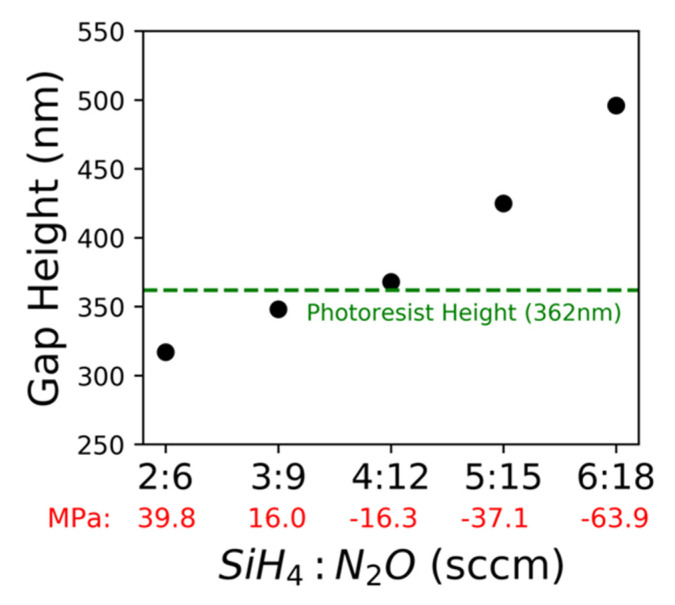
The gap height measured from the center of 20 µm-wide air-bridges is shown with respect to flowrate. The measurements were taken on a cross-section SEM image after the photoresist was removed. Initial photoresist height was also measured before removal. In red are the stress measurements corresponding to each of the flow rates.

The bridge shape changed from concave (tensile) to convex (compressive) with an increasing flowrate (Figure 6). While the gap height differences are difficult to see in the SEM images, they can be measured and plotted along with the corresponding flowrate and sacrificial photoresist height. Bridge height changed depending on flowrate, from concave to convex, (and, thus, intrinsic stress relaxation) as shown in Figure 6.

**Figure 7:**
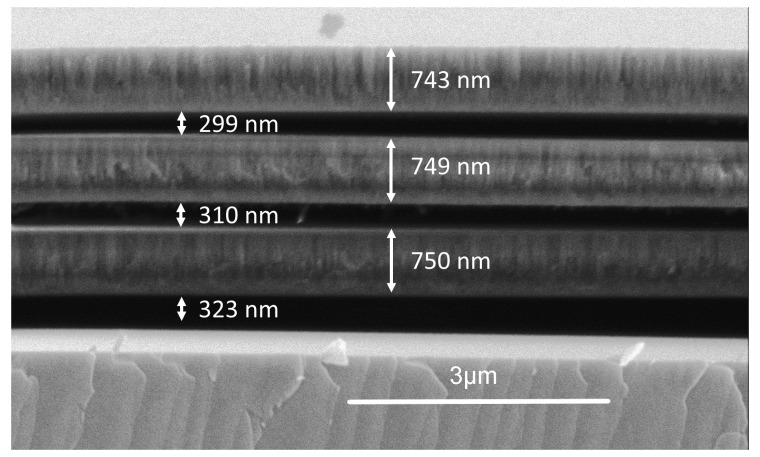
Three stacks of air-bridges were fabricated using the 4:12 sccm SiH_4_ to N_2_O ratio.

Stress relaxation direction of the films agrees with the measured stress of each film. The optimal depositional flowrate for minimal stress relaxation, where gap height is nearest to the sacrificial photoresist height, is 4:12 sccm SiH_4_:N_2_O. Air-bridge fabrication was successful using a 4:12 sccm flowrate on germanium and sapphire substrates, demonstrating the flexibility of air-bridge fabrication. Additionally, a stack of three SiO_2_ bridges was fabricated and imaged in Figure 7, further demonstrating the feasibility, durability, and flexibility of this fabrication process.

## 5. Conclusions

The gas flowrate was found to be the primary variable for creating a tunable parameter that allowed for the deposition of compressive and tensile SiO_2_ films without significantly impacting the refractive index of the film. To achieve a high tensile film, the deposition rate will be very slow. The greatest advantage of using this method is the flexibility to fabricate suspended SiO_2_ structures on different substrates or temperature-sensitive materials (e.g., Si, Ge, photoresist, SiO_2_, and Al_2_O_3_). The long-term stability of the air-bridges should be studied. Future research focus could include the fabrication of a simple photonic architecture, such as distributed Bragg reflectors or waveguides, for optical characterization in comparison to analytical models used to join UV emitters and photodiodes in a complete PIC.
